# CLOSING THE EQUITY GAP: DEMENTIA RESEARCH WITH INDIGENOUS POPULATIONS

**DOI:** 10.1093/geroni/igae098.0891

**Published:** 2024-12-31

**Authors:** Jordan Lewis, Kristen Jacklin, Jordan Lewis

**Affiliations:** Chair; Co-Chair; Discussant

## Abstract

This symposium highlights community-based work focusing on Alzheimer’s disease and related dementias (ADRD) conducted with Indigenous populations across the United States and Canada. We describe not only outcomes and findings of these various projects but also the essential research processes necessary for creating relationships and working with Indigenous partners and communities. We begin with Whetung, who takes a quantitative approach to describe the landscape of cognitive disparities among Indigenous older adults due to structural inequities, in particular, everyday and lifetime discrimination experiences. Next, Blind et al. describe the in-depth and iterative process of cultural adaptation of cognitive assessments. They are currently adapting validated cognitive assessments for Indigenous populations in Canada, this time for urban and reservation-residing American Indian populations. The final presentations by Jacklin et al. and Lewis will focus on qualitative research of cultural explanatory models and lived experience of dementia from the viewpoints of two different participant groups: healthy older adults (Indigenous communities in Minnesota, Wisconsin, and Ontario) and caregivers of people living with dementia (Alaska Native). ADRD research with Indigenous Peoples is scarce but growing, and this symposium highlights the innovate, various, and exceptional approaches to this research. This symposium highlights research projects which are rooted in the values of Indigenous knowledges and research methods, community-based participatory research, qualitative research, and the Six Rs of research (respect, relationship, representation, relevance, responsibility, reciprocity; Tsosie et al. 2022). Using these approaches, our research seeks to understand and close the equity gap for Indigenous older adults dealing with ADRD. Indigenous Peoples Interest Group Sponsored Symposium

